# Positioning of centrioles is a conserved readout of Frizzled planar cell polarity signalling

**DOI:** 10.1038/ncomms11135

**Published:** 2016-03-29

**Authors:** Jose Maria Carvajal-Gonzalez, Angel-Carlos Roman, Marek Mlodzik

**Affiliations:** 1 Department of Developmental & Regenerative Biology, Tisch Cancer Institute, and Graduate School of Biomedical Sciences, Icahn School of Medicine at Mount Sinai, One Gustave L. Levy Place, New York, New York 10029, USA; 2 Champalimaud Neuroscience Programme, Avenida de Brasilia, Lisbon 1400-038, Portugal

## Abstract

Planar cell polarity (PCP) signalling is a well-conserved developmental pathway regulating cellular orientation during development. An evolutionarily conserved pathway readout is not established and, moreover, it is thought that PCP mediated cellular responses are tissue-specific. A key PCP function in vertebrates is to regulate coordinated centriole/cilia positioning, a function that has not been associated with PCP in *Drosophila*. Here we report instructive input of Frizzled-PCP (Fz/PCP) signalling into polarized centriole positioning in *Drosophila* wings. We show that centrioles are polarized in pupal wing cells as a readout of PCP signalling, with both gain and loss-of-function Fz/PCP signalling affecting centriole polarization. Importantly, loss or gain of centrioles does not affect Fz/PCP establishment, implicating centriolar positioning as a conserved PCP-readout, likely downstream of PCP-regulated actin polymerization. Together with vertebrate data, these results suggest a unifying model of centriole/cilia positioning as a common downstream effect of PCP signalling from flies to mammals.

## Abstract (web summary)

Planar cell polarity (PCP) contributes to cellular orientation during development but how this is regulated in *Drosophila* is unclear. Here, the authors identify Frizzled-PCP signalling as regulating polarised centriole positioning in the wing disc.

## Graphical abstract

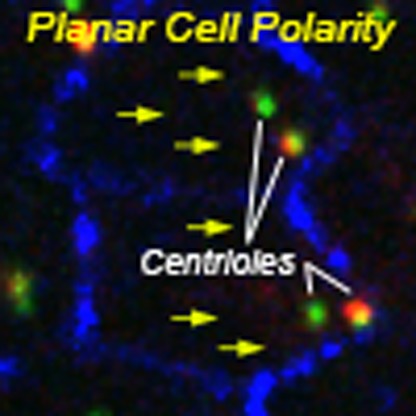

Polarized centriole positioning is important for properly oriented cell division, cilia positioning and cell migration. In mammals, planar polarized centriole postioning, as a part of the basal body of cilia, is necessary for proper directional beating of cilia at the apical surface within the node to establish left–right asymmetry, or in ependymal cells to promote cerebrospinal fluid circulation, among many other vital functions^1^,^2^,^3^,^4^,^5^. The coordination of cilia/centriole positioning from cell to cell across a tissue has been shown to be dependent on Frizzled/planar cell polarity (Fz/PCP) signalling in vertebrates^6^,^7^,^8^. Epithelial cells in *Drosophila*, on the other hand, do not have cilia and so the effect on cilia positioning versus general centriole positioning can be separated.

PCP refers to polarization within the epithelial plane and is regulated by two distinct and conserved protein pathways, the Fat/Dachsous pathway (Ft/Ds-PCP) and the core Fz pathway (Fz/PCP)^9^,^10^. The conserved mechanism(s) of Fz/PCP signalling are mediated by Wnts and two protein complexes, Fz/Dishevelled/Diego/Flamingo (Fz/Dsh/Dgo/Fmi (a.k.a. Starry night, Stan)) and Van Gogh/Prickle/Flamingo (Vang (a.k.a. Strabismus, Stbm)/Pk/Fmi), which localize to opposite junctional domains within each epithelial cell^11^,^12^,^13^,^14^,^15^,^16^,^17^, for example, to distal and proximal sides, respectively, in *Drosophila* wing cells. PCP signalling generally coordinates cell polarity across tissues, including ciliary positioning, the latter being reflected in the growing number of human diseases linked to aberrant Wnt-Fz/PCP pathways^18^. Ciliopathies, including Bardett–Biedl, Joubert and Meckel–Gruber syndromes, as well as neural tube closure defects in the embryo^19^,^20^, are linked to vital roles of PCP in cilia positioning and orientation, and directed cell movements during gastrulation^21^. There is growing evidence linking core components of the Fz/PCP pathway to ciliary positioning in vertebrates, that is, in the developing mouse embryo the basal body of node cilia shifts from the centre towards the posterior side of the node cells in a PCP-dependent manner^22^. In fact, Inversin, a vertebrate homologue of Dgo, localizes to the basal body and axoneme and is part of the NPHP (nephronophthisis) disease module, and its loss-of-function (LOF) alleles affect cilia morphogenesis, convergent extension, and left–right determination^23^. Vangl2 can also localize to the basal body and axoneme in ciliated cells^24^, and it affects the position and tilting of cilia^8^. Moreover, a Dishevelled triple knockout (*mDvl1, 2, 3*^*−/−*^) in multi-ciliated ependymal cells causes hydrocephalus and mis-positioning of cilia^25^, a phenotype similar to LOF of the mammalian Fmi homologues, Celsr2 and Celsr3 (refs 26, 27). Fz/PCP signalling controls ciliary positioning in all vertebrates examined, including mice, zebrafish and *Xenopus*^6^,^7^,^8^,^28^, and it controls intracellular positioning of centrioles during zebrafish gastrulation, biasing it towards the posterior cell region^29^. In other contexts, including non-ciliated epithelial cells in *Drosophila*, the effect of Fz/PCP signalling on ciliary components, including acetylated tubulin or centrioles, remains unknown.

It is thus an important evolutionary question whether Fz/PCP acts on centriole positioning in general, including in non-ciliated tissues where PCP signalling is ‘active'. We thus decided to explore centriole distribution and positioning in *Drosophila* wing epithelia, where the effects of PCP are well established, but which is a non-ciliated epithelium, as are all *Drosophila* imaginal disc epithelia. The *Drosophila* wing is one of the best-established tissues in which to study PCP pathways^11^,^12^,^13^,^14^,^15^,^16^,^17^. Adult wings manifest PCP with a single distally pointing actin-based hair in each cell (a trichome)^30^. At the pupal stage, when the wing is formed by two monolayers of non-ciliated epithelial cells juxtaposed at their basal membranes, at around 30–32 h APF, the trichomes start to appear, as actin polymerization becomes activated and focused at the distal apical vertex of each cell. This process depends on Rho family GTPases, which are recruited and activated by Fz-Dsh/PCP complexes^31^,^32^,^33^. Microtubules (MTs) also change in arrangement, from a radial to parallel distribution, projecting towards the distal apical portion of the cell with a distal bias of MT plus ends^34^,^35^,^36^. Although Fz/PCP signalling induces changes to the cytoskeleton, many unanswered questions remain how PCP regulates cytoskeletal elements, and it is for example unknown what type of MTs are involved in actin-hair formation in pupal wings and if these are linked to actin polymerization or centriole positioning among other options.

In this study, we demonstrate that in non-ciliated cells of the *Drosophila* wing the positioning of centrioles is polarized towards the Fz/Dsh side of each cell and, importantly, under the control of the core Fz/PCP system. Our *in vivo* data in *Drosophila* wings argue for and provide evidence that centriole positioning is a conserved PCP readout, likely shared in all epithelial cells.

## Results

### Centriole polarization in pupal wing cells

**Figure 1:**
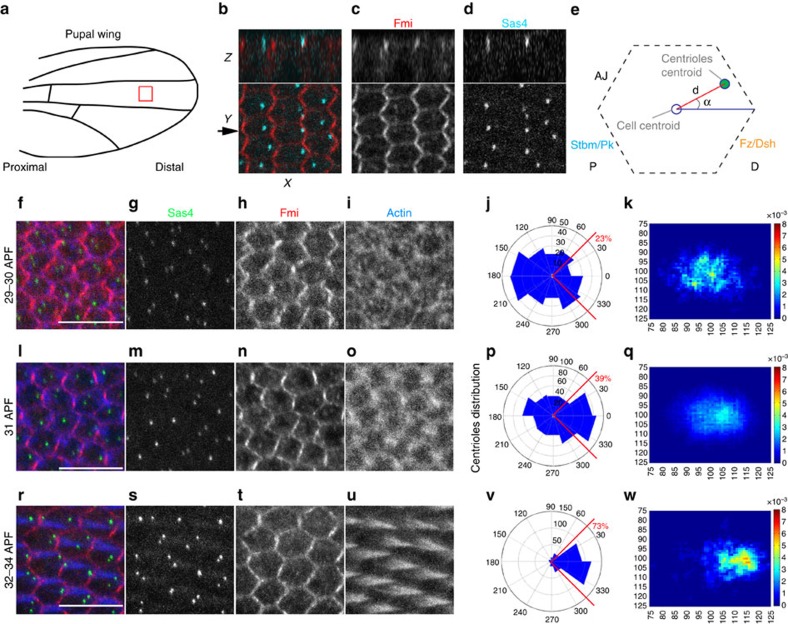
Centriole localization and positioning during PCP establishment in pupal wings. (**a**) Illustration of pupal wing and its orientation (**b**–**d**) Sas4-labelled centrioles (cyan in **b** and monochrome **d**) are distributed in the junctional planes (*X*–*Z* plane—upper panels) (marked by Fmi staining, red in **b**, monochrome in **c**). Top panels are *x*–*z* sections of respective *x*–*y* views shown below. Scale bar, 10 mm. (**e**) Schematic representation of a pupal wing epithelial cell and the parameters used to study centriole positioning. (**f**–**w**) During pupal wing development, centriole localization changes. (**f**,**l**,**r**) Sas4 (green), Fmi stained in red, and actin (phalloidin) in blue, and the respective monochromes. (**f**–**k**) Before hair formation (29 h APF), centrioles are unpolarized in a central position in the apical portion of each cell (quantified in **f**,**k**). (**l**–**q**) At the onset of hair formation (31 h APF), centrioles begin to localize to the distal portion of each cell (quantified in **p**,**q**). (**r**–**w**) Subsequently, when hairs are fully present in all wing cells (32–34 h APF), centrioles appear to be polarized mostly to the distal sector of each cell (quantified in **v**,**w**). Scale bar, 10 mm. Red sectors in **f**,**p**,**v**—% within distal quadrant. Statistical analyses: rosette diagram distributions panel **g** versus **p**: *P<*0.0001; **p** versus **v**: *P<*0.0001 (χ^2^-test).

Using non-ciliated cells in imaginal discs, we asked whether centriole positioning is linked to Fz/PCP signalling as an evolutionarily conserved readout of Fz/PCP establishment (or if the presence of cilia is a pre-requisite for a Fz/PCP signalling-centriole connection). We first established a quantitative method to assess centriole positioning during pupal wing development, at the time when cytoskeletal rearrangements are being established downstream of Fz/PCP signalling, establishing a distally oriented trichome/actin hair. Two core centriolar components, Sas4 and Asterless (Asl), serve as excellent markers for centrioles; Sas4 and Asl, which is a centriolar scaffold required for centriolar assembly^37^. We analysed the localization of centrioles, via Sas4 and Asl staining, in pupal wing epithelial cells relative to other cellular markers, leading to two initial general observations on centriolar positioning: (1) centrioles are localized apically in cells at the level of the adherens junctions (Fig. 1); and (2) Centriole positioning became progressively more polarized and localized to the distal vertex of each cell (Fig. 1 and Supplementary Fig. 2). Centrioles were detected at the adherens junction level, which were labelled with Fmi, and were never detected more basally (for example, at the level of Dlg/Discs large, a marker for baso-lateral membrane^38^; Fig. 1 and Supplementary Fig. 1). This apical localization is very similar to that in vertebrate polarized epithelial cells. As the cells matured and started to display polarized, distal actin polymerization (phalloidin staining; Fig. 1), centrioles became also localized to the distal vertex of each cell (Fig. 1 and Supplementary Fig. 2).

To characterize the timing of centriole re-positioning in pupal wing epithelial cells, we determined centriole positions before and during hair formation relative to the centre of the cell, measuring distance and angles between the centroid of the cell and the centroid of the centriole in individual cells (Fig. 1f–w; see Methods and Supplementary Fig. 2 for technical details). Before the actin-based hair is formed (28–30 APF), centrioles are positioned near randomly but close to the centroid of the cell (Fig. 1f–i; see quantification in Fig. 1j–k), and can be found at any angle (rosette diagram in Fig. 1j; also Supplementary Fig. 2). At 31 APF when actin starts to be enriched at distal vertex, centrioles appear biased towards the distal cellular vertex, the distal quadrant between −45° and +45° (Fig. 1l–q, compared with non-polarized localization at 29–30 APF, Figure 1f–k). Once hairs are detected in all cells (32–33 APF), centriole localization is fully polarized to the distal vertex of cells (relative to the wing margin) (Fig. 1r–w). This subcellular distribution of centrioles is very similar to that of the actin-based hair centroids (Supplementary Fig. 2). Taken together, we conclude that centriole positioning becomes planar polarized in non-ciliated epithelial cells, following largely the same distribution as the trichomes, the actin-based hairs and the best defined ‘cellular effect' of Fz/PCP signalling in *Drosophila* wings.

### PCP signalling instructs centriole positioning

**Figure 2:**
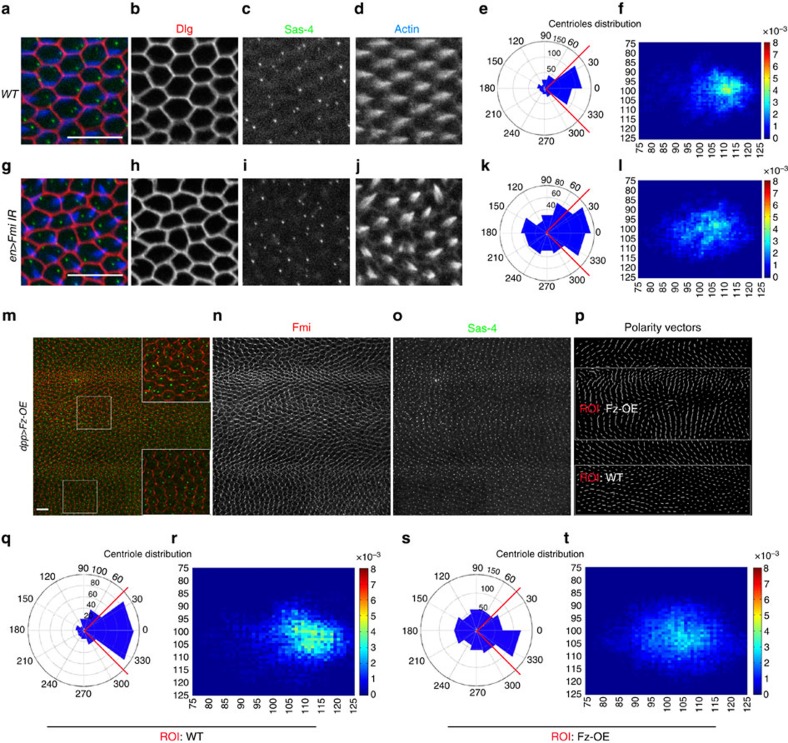
Centriole position is affected by PCP signalling. (**a**–**l**) Fmi LOF, using *en*-driven *fmi-IR* knockdown affects centriole localization; (**a**,**g**) Dlg: red, cell outline; Sas4: green, centriole; actin (phalloidin): blue; and respective monochromes in **a**–**d**,**h**–**j**. Centrioles within cells in the *en>fmi-IR* area (**g**) are less polarized and are distributed more centrally, quantified in **e**–**f** and **k**–**l**, respectively. (**m**–**t**) Fz gain-of-function (GOF; see Methods), causes Fmi depolarization (see polarity vectors in **p**), and defects in centriole distribution (Fmi in red, monochrome in **n**; Sas4 in green, monochrome in **o**). Fz overexpressing cells have central distribution of centrioles (quantified in **s**–**t**; see also ROI: Fz-OE in **p**), as compared with *WT* areas of *dpp>Fz-OE* wings (quantified in **h**,**i**; see ROI: *WT* in **p**). Scale bar, 10 μm. Statistical analyses: centriole rosette diagrams, **e** versus **k**: *P<*0.0001; **q** versus **s**: *P<*0.0001 (χ^2^-test).

As PCP signalling regulates wing hair (trichome) formation in *Drosophila* and the position of basal bodies (cilia) in vertebrates, we next investigated whether Fz/PCP regulates the position of centrioles during pupal wing development. We characterized the positioning of the centrioles in loss or gain-of-function core Fz/PCP pathway backgrounds, with the same quantitative approach described above, using *en-Gal4* driven *fmi-IR* (dsRNA knockdown; see Methods) and *fz* null allele (*fz*^*P21*^) wings (Fig. 2, and Supplementary Fig. 3) and *dpp*-driven Fz overexpression (Fz-OE; GOF) background. In both genetic scenarios, centriole positioning was altered (Fig. 2 and Supplementary Fig. 3). Although apical localization was maintained, centriole positioning remained unpolarized and more centered within mutant cells in both cases (cf. heat maps in Fig. 2f,l,r,t), consistent with randomized PCP in each case. Moreover, angle distribution was spread over a much wider range of angles with WT regions of same wings remaining polarized and serving as controls (compare rosette diagrams in Fig. 2e,k,q,s, respectively; see also Supplementary Fig. 3). These data are consistent with the notion that in epithelial cells centriole positioning is generally connected to Fz/PCP signalling.

### Cytoskeleton and centriole localization are linked

**Figure 3:**
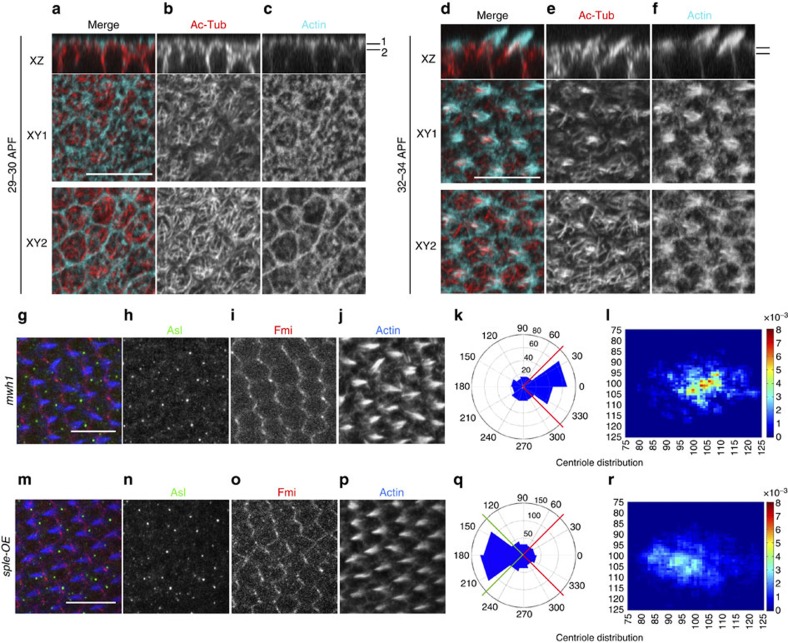
Centriole localization relative to trichome position. (**a**–**c**) *XZ* and two *XY* optical sections of same wing area, showing polymerized actin (phalloidin, cyan) and acetylated tubulin staining (red, monochrome in **b**). At 29 h APF before actin-based hairs are formed, both actin and acetylated tubulin appear enriched at the apical portion of the pupal wing cells; two planes, apical (*XY1*) and subapical (*XY2*) are shown in the *XY*-axis (indicated in the *XZ* sections as black lines 1 and 2). (**d**–**f**) After hair formation (32 h APF), acetylated tubulin is enriched at the base of each hair and within the polymerized actin structure. *XZ* and two *XY* planes, apical (*XY1*) and subapical (*XY2*) are shown in the *XY*-axis from the same; stainings as in **a**. (**g**–**l**) Centriole localization in the *mwh*^*1*^ mutant is less polarized than *wt* (in 32 h pupal wing cells; compared with Fig. 1v,w). Asl (green), Fmi (red) and phalloidin (blue) stainings and the respective monochromes are shown. **k** and **l** show centriole distribution quantifications in *mwh*^*1*^ mutant, note shift to less polarized, central distribution most evident in heat map (**l**). (**m**–**q**) Overexpression of Sple (Sple-OE) causes reversal of cellular polarity and hair position, and accordingly also centriole positioning is inverted; quantifications in **q**,**r**. Asl staining position was used for quantifications in rosettes (**k**,**q**) and heat maps (**l**,**r**). Scale bar, 10 μm. Statistical analysis: centriole rosette diagrams, **k** versus Fig. 1w (*wt* control): *P<*0.001; **q** versus *wt* control: *P<*0.0001 (χ^2^-test).

To determine how centriole positioning in pupal wing cells relates to the cytoskeleton, we next analysed both MTs and actin in pupal wing cells. MT localization and actin have been previously stained during PCP establishment in pupal wings, using confocal and electron microscopy^32^,^34^,^39^, but the nature of the MTs remained unexplored. We tested whether they were acetylated, a marker of stable MTs, since acetylated MTs are generally associated with cilia and the basal body in ciliated cells. In pupal wings before hair formation, actin was enriched in the apical plane of the cell and acetylated MTs also formed a web-like structure in the apical plane of each cell (Fig. 3a–c). Once actin-based hairs started to form and actin polymerization was focused at the distal vertex of the apical membrane, acetylated MTs became enriched at the base of the hairs (Fig. 3d–f). Moreover, during hair formation, acetylated MTs started to ‘invade' the hair itself, forming what looks like a scaffold-structure for the trichome (Fig. 3d–f). Of note, the juxtaposition between actin and acetylated tubulin was maintained into deeper areas in the cell, not just in the most apical planes from which the hair projects (Supplementary Fig. 4). When centrioles were co-labelled with actin in the presence of the hair structure/trichome, they were localized adjacent to the base of the hair (co-labelled with actin; Supplementary Fig. 5).

The close co-localization of centrioles, acetylated tubulin and actin raises the question of whether they remain connected or become disconnected in PCP LOF or GOF backgrounds. Knockdown of *fmi* in the posterior wing compartment causes PCP defects, reflected in aberrant hair orientation and some multiple cellular hair (mch) defects (Fig. 2g and Supplementary Fig. 3i). Generally in mutant PCP backgrounds, the actin hair in each cell appears unpolarized (within the apical membrane plane), often near the centre of a cell. When acetylated tubulin was examined in *fmi*-IR conditions, it remained associated with the growing actin hair, independently of where the hair growth was positioned within the cell. Furthermore, this link between acetylated tubulin, centrioles and actin remained in GOF scenarios, for example, when Fz was over-expressed (for example in the *dpp* stripe of 8–14 cells close to vein L3; Supplementary Fig. 6). These results suggested that acetylated tubulin/ MTs are a structural component of the actin-based hair and that the link between actin and acetylated tubulin is independent (or downstream) of Fz/PCP establishment, even though the position of the structure within the cell is regulated by Fz/PCP activity.

Next we asked whether the number of actin hairs or hair positioning and thus actin polymerization downstream of Fz/PCP could affect centriole localization. The null allele of multiple wing hair (*mwh*^*1*^) shows mch formation. Mwh, becomes localized in response to Fz/PCP factor interactions and acts downstream of the Fz/PCP effectors Inturned and Fuzzy, and it is thought to directly affect actin polymerization. Moreover, it does not affect localization of the core Fz/PCP factors (for example, Fmi or Fz (refs 40, 41)). Centriole localization was less polarized in *mwh* mutants (Fig. 3g–j; see heatmap in Fig. 3e, although angular distribution was less affected when compared with *fz* or *fmi* LOF and GOF backgrounds; for example, compare Figs 3k and 2k,s). These data suggest that centriole positioning is a downstream event of localized actin polymerization, which in turn is regulated by Fz/PCP. Over-expression of the Sple isoform of *pk* in developing wings has recently been reported to reverse PCP orientation, resulting in actin hair formation being moved to the proximal cellular vertex without affecting Vang or Dsh localization^36^,^42^. Importantly, Sple-OE (see Methods) caused not only a reversal of actin hair polymerization but also a reversal of centriole positioning, with the majority of centrioles located to the proximal vertex (Fig. 3m–r). Taken together, these data are consistent with the model that centriole positioning is a downstream readout of core Fz/PCP signalling, similarly to properly localized actin polymerization, and, more importantly (based on the *mwh* LOF effects) that centriole positioning is a downstream effect of localized actin polymerization as regulated by the core Fz/PCP pathway.

### Loss or gain of centrioles does not affect PCP

**Figure 4:**
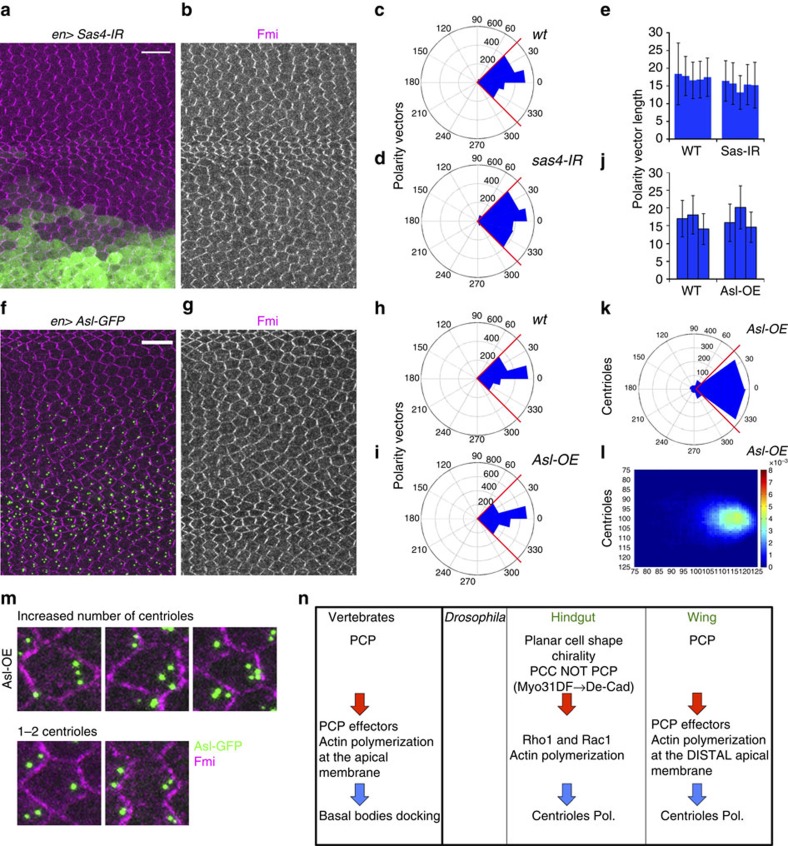
Loss or gain of centrioles does not affect core PCP factor localization. (**a**–**e**) Imaging and quantifications (sed Methods) of Fmi staining in *Sas4* RNAi-mediated knockdown (in cells marked with GFP) in the posterior compartment in 32–34 h APF pupal wings. *en>Sas4*^*IR*^ RNAi-mediated KD (**a**–**b**) did not disrupt Fmi localization as compared with the WT anterior compartment from same wings. (**c**,**d**) Quantifications of Fmi polarization using polarity vector angle orientation revealed no significant differences between *en>Sas4*^*IR*^ (*n*=1,872 cells from five independent wings) and WT cells (*n*=1,513 cells, five independent wings). NS: non-significant (*P=*0.655) (χ^2^-test)). (**e**) Polarity vector lengths (relative to Fmi fluorescence polarization) did not show significant differences between in *Sas4*^*IR*^ cells and adjacent WT cells (five independent wings). NS: non-significant. (**f**–**j**) Gain of centrioles (>2 per cell) through Asl overexpression (*n*=1,099 cells, three independent wings) in the posterior compartment (*en>Asl*), did not affect Fmi localization as compared with wild type from the same wings (*n*=1,096 cells, three independent wings). (**h**,**i**) Quantification of angle distribution of polarity vectors, and (**j**) polarity vector length. Note no significant changes between *wt* and *en>Asl* cells (*P=*0.783) (χ^2^-test). (**k**–**m**) Centriole positioning follows PCP core factor localization even when higher numbers of Asl-positive centrioles are present per cell. Quantifications are depicted in rosette (**k**) and heatmap (**l**) diagrams. (**m**,**n**) Specific examples of centriole localization in multi-centriolar cells, note several examples with 3–4 centrioles per cell (**m**, top), and two Asl-positive centrioles per cell (**m**, bottom); in *WT* only 1 centriole is stained by Asl for comparison (Asl in green; cf. to Fig. 1a,b). In all cases, centrioles remained close to the distal side of each cell (marked with Fmi; magenta). (**n**) Schematic representation of signalling pathways involved in centriole polarization related to planar cell polarity in vertebrates versus *Drosophila*. Scale bar, 10 μm.

We next asked if loss or gain of centrioles could affect core PCP factor localization. Importantly, loss of centrioles, via Sas4 knockdown or the *sas4*^*S2214*^ null allele, did not result in detectable PCP phenotypes in pupal wing cells when Fmi staining/polarization was assessed (Fig. 4a–e and Supplementary Fig. 8), although growth defects were observed as recently reported (Supplementary Fig. 8; surprisingly, flies lacking Asl or Sas4, and thus centrioles, survive to adulthood due to compensatory cell proliferation; their adult wings exhibit blisters, overgrowth, and vein mis-patterning^43^,^44^,^45^). In contrast, Asl and Sak/Plk4 (Polo-like kinase 4) overexpression caused an increase in centriole number^37^,^46^, for example under *en-Gal4* conditions (*en>Asl*; Fig. 4f,m) or ubiquitously (Sak/Plk4, expressed via *ubiquitin* promoter; not shown). In such conditions, we did not detect defects in Fmi localization at the pupal stage (Fig. 4f–l), with respect to either core PCP generated nematic order, actin hair orientation or centriole positioning (see also Supplementary Fig. 7). Strikingly, however, in the thus generated multi-centriolar cells, when more than two centrioles were present (Asl normally only labels one centriole per cell, the mother centriole; Supplementary Fig. 1), all centrioles were positioned near the distal vertex (Fig. 4k–m), and moreover were always located near the base of the hair (Supplementary Fig. 7). These results indicate that the number of centrioles does not impact Fz/PCP signalling and further confirm the notion that centriole positioning is a downstream effect of core Fz/PCP signalling via its effects on actin polymerization.

## Discussion

Taken together with observations that Fz/PCP signalling regulates basal body and cilia positioning in vertebrates^6^,^7^,^8^,^28^, our data on centriole positioning as a Fz/PCP readout in non-ciliated *Drosophila* wing cells indicate that centriole/MTOC (MT organizing centre)/basal body positioning is an evolutionarily conserved downstream effect of Fz/PCP signalling. Its link with actin polymerization (hair formation in *Drosophila* wing cells) suggests that actin polymerization effectors also affect cilia positioning, possibly through docking of the basal bodies to the apical membranes. Inturned, Fuzzy and Rho GTPases regulate apical actin assembly necessary for the docking of basal bodies to the apical membrane^47^,^48^ and this apical actin membrane accumulation is lost in Dvl1-3-depleted cells^47^,^48^,^49^,^50^.

In left–right asymmetry establishment of the *Drosophila* hindgut, which is not a Fz/PCP-dependent process^51^, asymmetric centriole positioning is observed. During this so-called planar cell shape chirality process, which affects gut-looping and thus embryonic left/right asymmetry, centriole positioning is however still dependent upon actin polymerization downstream of Rho GTPases (Rac and Rho), via MyoD and DE-cadherin control^51^,^52^. As Rho GTPases (Rac, Cdc42 and Rho) are downstream effectors of Fz-Dsh/PCP complexes, and their mutants cause PCP-like phenotypes including mchs or loss of hairs in wing cells^31^,^32^,^53^. It is thus tempting to infer that both processes, planar cell shape chirality and Fz/PCP, regulate centriole positioning through a common Rho GTPase-mediated actin polymerization pathway, initiated by an upstream cellular communication system, although this assumption will require experimental confirmation. In the mouse, Fz/PCP signalling regulates cilia movement/positioning in cochlear sensory cells via Rho GTPase-mediated processes^54^, suggesting a similar mechanism in a representative mammalian PCP model system (Fig. 4n). In conclusion, the positioning of centrioles appears to be a key and an evolutionary conserved downstream readout of Fz/PCP signalling, ranging from flies to mammals in both ciliated and non-ciliated cells.

## Methods

### Fly strains

Flies were raised on standard medium and maintained at 25 °C, unless otherwise indicated. GAL4/UAS system^55^ was used for gene expression and RNAi studies. The Gal4 expression drivers were *en-GAL4, dpp-GAL4 and nub-GAL4*. In addition the following lines were used: *fmi* RNAi (Mlodzik lab stock, ML117 *(2)*), Sas4 RNAi (KK106051 from VDRC and BL35049 from Bloomington Stock Centre), Sple-OE (gift from Masakazu Yamazaki; fly ID: TID29239 ref 42), GFP-Asl-OE, Sas4-GFP, *sas4*^*S2214*^ (gift from Jordan Raff), *fz*^*p21*^, *mwh*^*1*^ (described in Flybase), UAS-Fz for Fz-OE experiments^56^.

The different *Gal4* lines applied in this study were used to direct expression of the UAS-constructs to distinct wing compartments, linked to the localized expression of developmental genes such *engrailed (en)*, restricted to the posterior compartment of the wing, *decapentaplegic (dpp*), expressed in a stripe between L3 and L4, and *nubbin (nub)* expressed in the whole wing.

### Immunohistochemistry and immunocytochemistry

White pupae (prepupae) were collected and staged at 25 °C for indicated time points. Wings were dissected in PBS with 0.1% Triton X-100 (PBT) and fixed with 4% formaldehyde for 45 min at room temperature. Pupae were then washed twice in PBT and blocked in PBT with 2% bovine serum albumin for 30–45 min. Samples were incubated with primary antibodies overnight at 4° in PBT-0.2% bovine serum albumin. Samples were washed five times in PBT and incubated for 1 h in fluorescent secondary antibodies diluted in PBT and fluorescent phalloidin when indicated. Five additional washes in PBT were performed before pupal wings were detached from the pupal cage and mounted on slides with Vectashield (Vector Laboratories). Pupal wing images were acquired using a confocal microscope (× 40∼oil immersion, 1.4 NA; SP5 DM; Leica) with LAS AF (Leica) software.

The following antibodies were used: Anti-Asl (gift from Jordan Raff); anti-Fmi (from DSHB); anti-Dlg (gift from Kuyng-Ok Cho); anti-Cnn (gift from Jordan Raff); and acetylated-tubulin (from Sigma). Secondary antibodies used at 1:200 were from Invitrogen with different Alexa fluorophores (Alexa 568 and Alexa 647). FITC-phalloidin, rhodamine-phalloidin and Alexa 647-phalloidin were used at 1:500–1,000 (Invitrogen).

### Quantitative analyses of centriole positioning

A novel function in MATLAB was developed to assess the relative position of centrioles within cells using immunohistochemical images. As input, this function uses (i) an immunofluorescent image of centrioles marked by Sas4 or Asl and (ii) the same image, marked by Fmi or Dlg and processed with the software ‘packing_analyzer_V2' to obtain the sketches of the cell borders. First, each cell is automatically detected and its centroid (*R*) is calculated as the center of the mass of the cell, whose Cartesian coordinates are


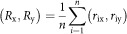


where (*r*_ix_, *r*_iy_) are the Cartesian coordinates of the pixel *r*_i_, and n represents the sum of pixels in the cell. Then, the centrioles present within the cell are recognized and, again, the centroid (*C* )representing the center of mass of each one is obtained as


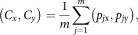


where (*p*_*j*x_, *p*_*j*y_) are the coordinates of the pixel *p*_*j*_, and *m* represents the sum of pixels in the centriole. The angle (*α*) formed between the centroid of the cell and the centroid of each centriole is calculated as


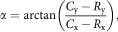


with a correction in the orientation for angle detection in the 0<=*α*<=2*π* radians range. The final results from a full set of cells were plotted as a rosette diagram representing these angles, and χ^2^-tests were used to detect significant differences between angle distributions (see figure legends).

Another representation of the relative centriole positions inside the cells was performed as follows: A general cell model for a specific genotype was generated as a 51 × 51 matrix called *M*. Then, each pixel *p* of each centriole was assigned to an element *E* in the matrix following the equation:


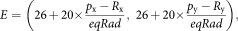


rounding each element to the nearest integer. *eqRad* represents the equivalent radius of the cell (the radius of a circle with the same area as the cell), and calculated as


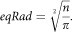


The final cell model is obtained as the density histogram of *M* using the whole set of pixels of each centriole, and represented as a colour heatmap. This function is available on the website http://www.neural-circuits.org/other-software, and can be applied to the relative location of other subcellular structures, like the actin hair.

### Quantitative analyses of polarized Fmi localization

Polarity as determined from anti-Fmi stained cells was calculated with the software ‘packing_analyzer_V2' as described in ref. 57. The software calculated both angles and strength of polarization (nematic order)^57^. Rosette figures were generated to represent the 360° orientation of the nematic order/polarity vector population, with 0° always being oriented as pointing distally using MATLAB. Statistical tests were used to assess differences between cellular orientation distributions (χ^2^-test) or polarity strength/nematic order (*t*-test).

## Additional information

**How to cite this article:** Carvajal-Gonzalez, J. M. *et al*. Positioning of centrioles is a conserved readout of Frizzled planar cell polarity signalling. *Nat. Commun.* 7:11135 doi: 10.1038/ncomms11135 (2016).

## Supplementary Material

Supplementary Information Supplementary Figures 1-8
