# Supplementary material for: Positioning of centrioles is a conserved readout of Frizzled planar cell polarity signalling
Source: Nat Commun. 2016 Mar 29;7:11135. doi: 10.1038/ncomms11135 (PMC4820615; doi:10.1038/ncomms11135)
Supplement: Supplementary Information — Supplementary Figures 1-8 [file ncomms11135-s1.pdf]

Supplementary Figure 1: Mother and daughter centrioles detection in pupal wings

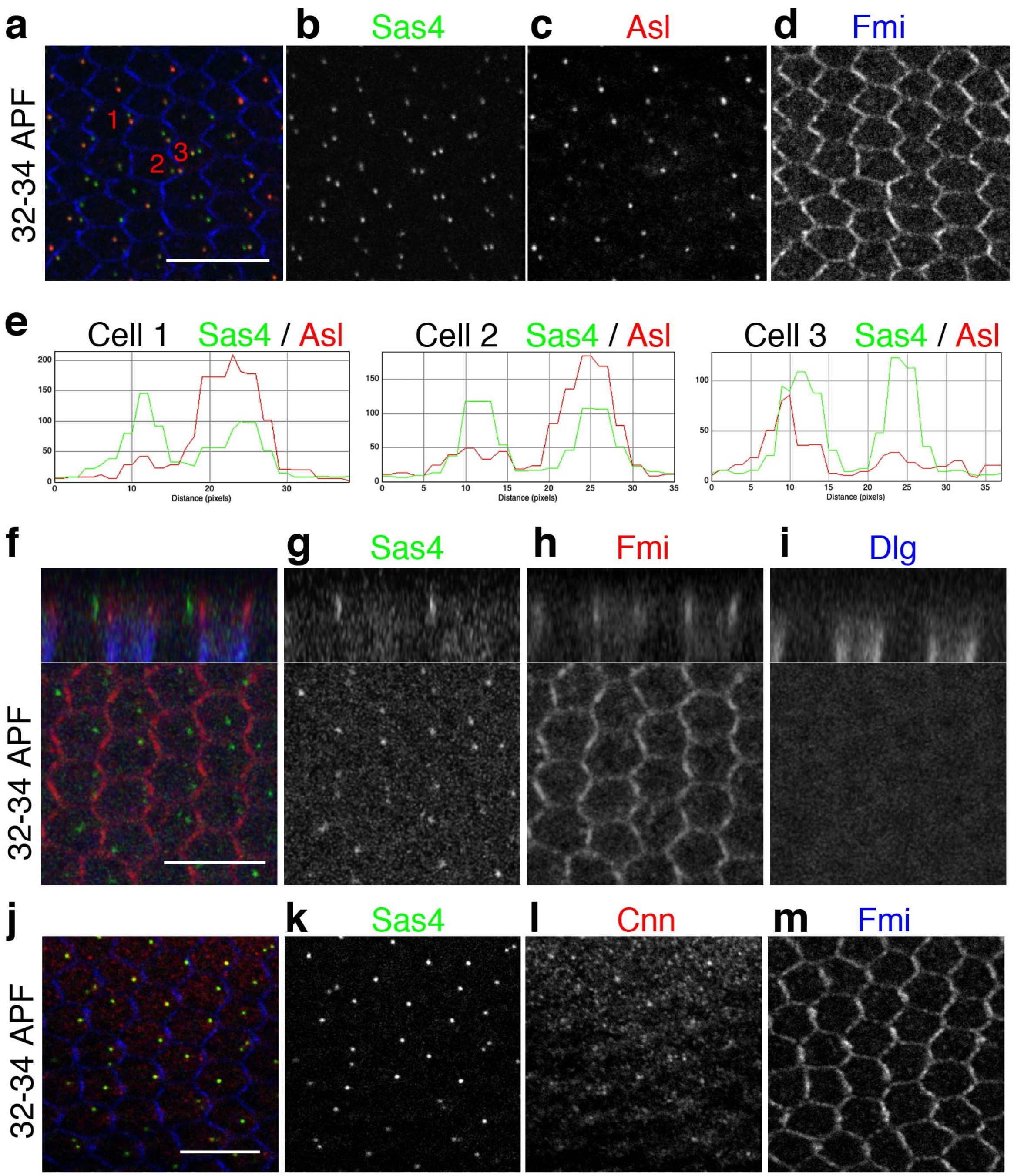

(**a-d**) Centrioles labeled with Sas4 (monochrome in **b**) and Asl (monochrome in **c**) in pupal wing epithelial cells from *wild-type Drosophila* wings. Fmi staining (**d**) serves as a PCP marker, displaying cellular polarity and proximal distal orientation. Scale bars represent 10µm

(**e**) Line scanning profiles for three cells with Asl in red and Sas in green.

(**f-i**) Co-staining of centrioles (Sas4 in green; monochrome in **g**), junctional Fmi (in red; monochrome in **h**),) and the basolateral marker Dlg (in blue; monochrome in **i**) in *wildtype* pupal wings. Top panels are x-z sections of respective x-y views shown below. Scale bars represent 10μm.

(**j-m**) Co-staining of centrioles (Sas4 in green; monochrome in **k**), the pericentriolar marker centrosomin (Cnn in red; monochrome in **l**), and junctional Fmi (in blue; monochrome in **m**) in *wild-type* pupal wings. Scale bars represent 10μm.

Supplementary Figure 2: Centrioles polarization quantification

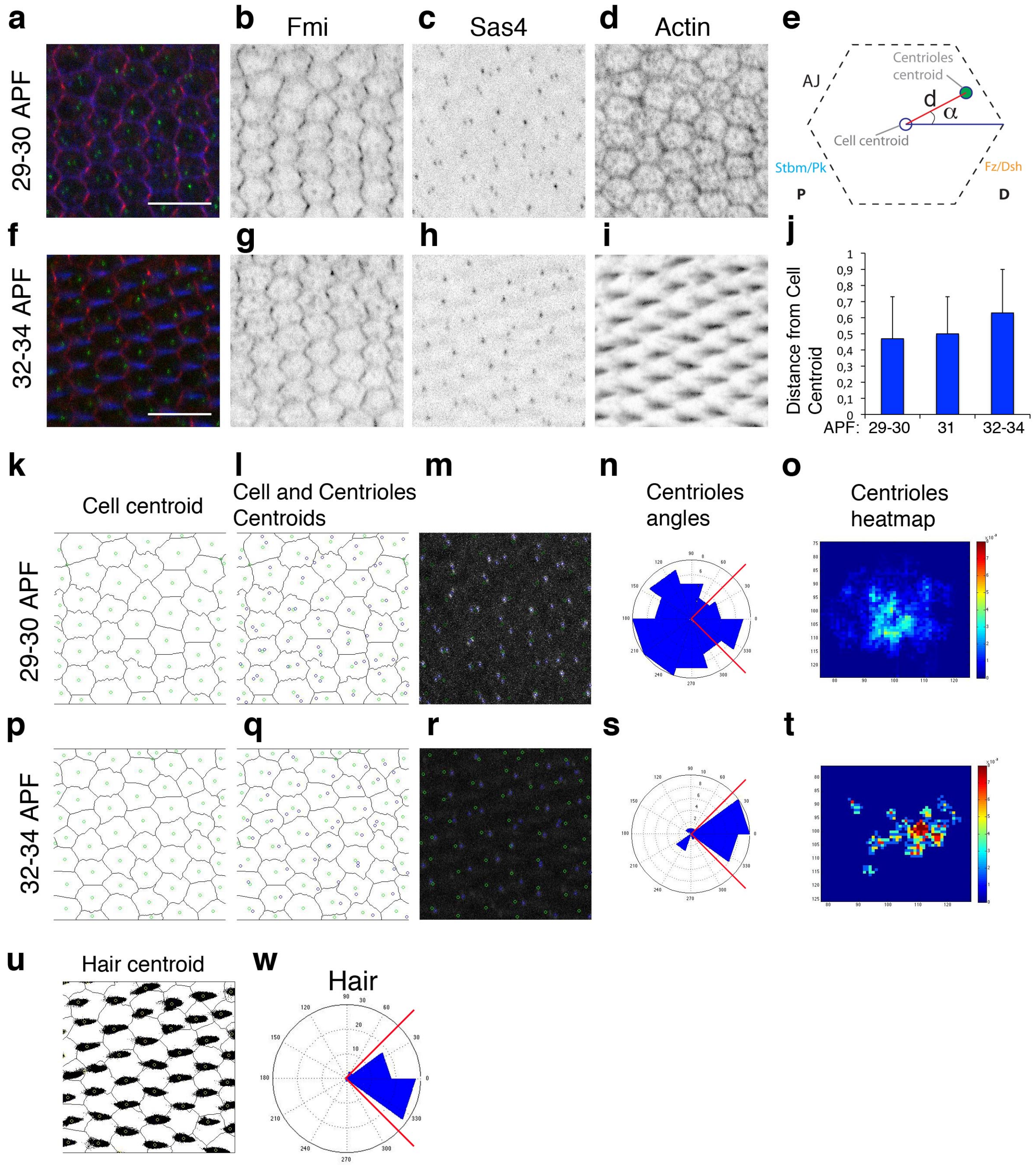

(**a-d**) Co-staining of centrioles (Sas4 in green; monochrome channel in **c**), the junctional marker Fmi (in red; monochrome channel in **b**),) and actin labeled with Phalloidin (in blue; monochrome in **d**) before start of hair formation (29-30h APF).

(**e**) Schematic representation of an epithelial cell at the junctional level and the parameters used to study centriole positioning**.**

(**f-i**) Co-staining of centrioles (Sas4 in green; monochrome channel in **h**), the junctional marker Fmi (in red; monochrome channel in **g**),) and the actin based hairs labeled with phalloidin (in blue; monochrome channel in **i**) once the hairs are present (32-34 APF). Scale bars represent 10μm.

(**j**) Quantification of the average distance of the centriole from the cell centroid; note increasing distance as wing cells develop, with a significant difference ($p<0.05$) between 29-30APF and 32-34APF (as determined with a *t*-test).

(**k-o**) Sequential steps in the quantitative analysis of centroid relative location for 29-30h APF, with cell centroid detection (in green; **k**), centriole centroid position (in blue; **l**), and merged with Sas4 monochrome channel (**m**); the rosette of the angles between cell and centriole detection (**n**) and the centriole density in a cell model (**o**) are the representations of the relative position of centrioles in a set of cells.

(**p-t**) Sequential steps in the quantitative analysis of centroid relative location for 32-34h APF, with cell centroid detection (in green; **p**), centriole centroid position (in blue; **q**), and merged with Sas4 monochrome channel (**r**); the rosette of the angles between cell and centriole detection (**s**) and the centriole density in a cell model (**t**) are the representations of the relative position of centrioles in a set of cells.

(**u-w**) Actin-hair base centroid positioning relative to the cell centroid, established using thesame quantification parameters as for centrioles.

Supplementary Figure 3: Centrioles localization phenotypes in PCP GOF and LOF

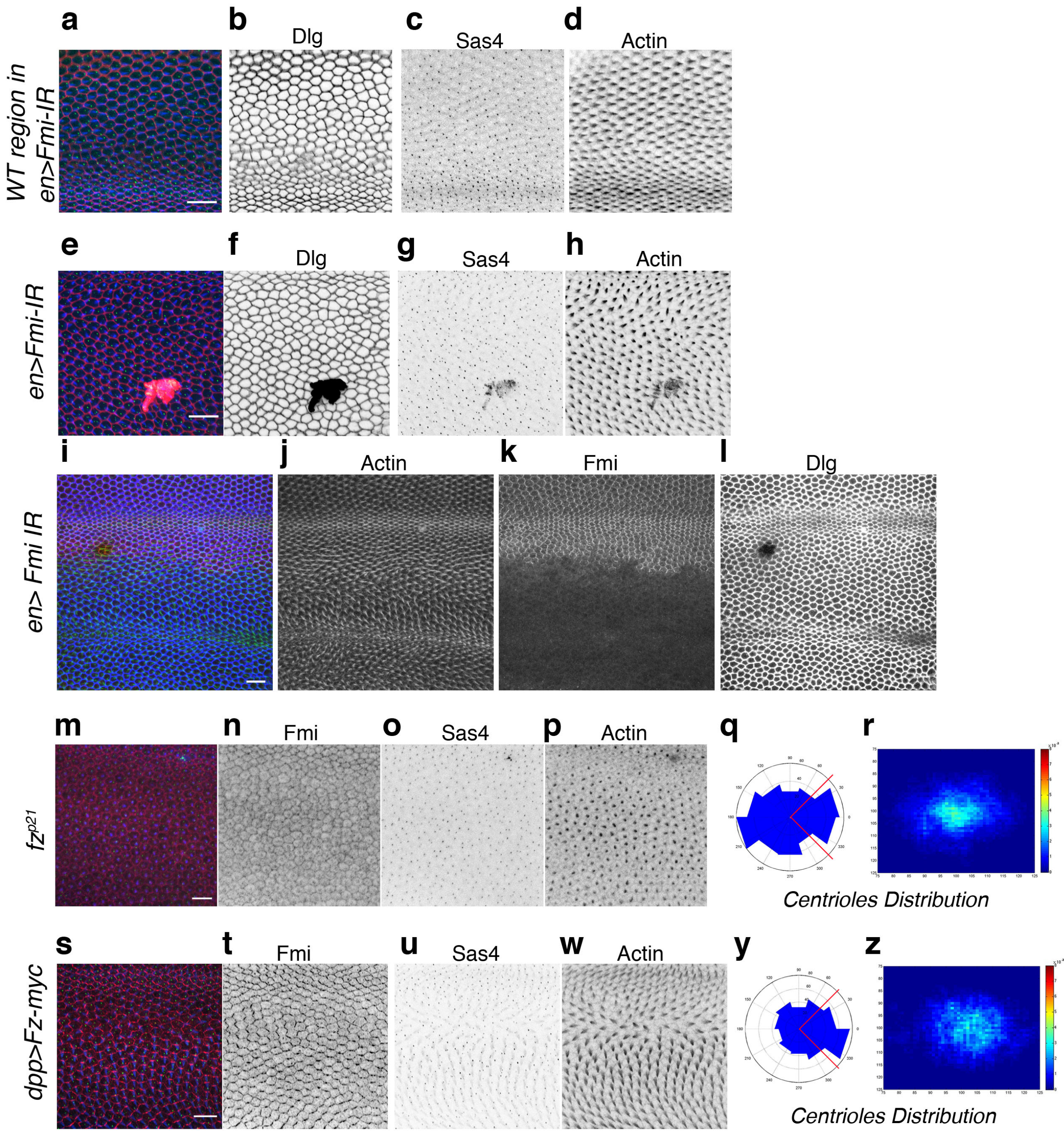

(**a-d**) Co-staining of centrioles (Sas4 in green; monochrome channel in **c**), the basolateral marker Dlg (in red; monochrome channel in **b**),) and the actin based hairs labeled with phalloidin (in blue; monochrome channel in **d**) in the anterior domain (WT domain) of *en>Fmi IR* pupal wing.

(**e-h**) Co-staining of centrioles (Sas4 in green; monochrome channel in **g**), the basolateral marker Dlg (in red; monochrome channel in **f**),) and the actin based hairs labeled with phallodin (in blue; monochrome channel in **h**) in the posterior domain (Fmi

knock-down)of an *en>Fmi IR* pupal wing.

(**i-l**) Knock-down efficiency for Fmi in the posterior compartment of the pupal wing. Costaining of the actin based hairs - Phalloidin (in green; monochrome channel in **j**), the basolateral marker Dlg (in blue; monochrome channel in **l**), and Fmi (in red; monochrome channel in **k**). The image shows a region spanning the anterior (*WT*) – posterior (knock-down) border. Scale bars represent 10µm.

(**m-p**) Co-staining of centrioles (Sas4 in green; monochrome channel in **o**), junctional marker Fmi (in red; monochrome channel in **n**) and the actin based hairs labeled with phallodin (in blue; monochrome channel in **p**) in a homozygous fz_{p21} null pupal wing. Scale bars represent 10µm.

(**q-r**) Centriole distribution quantifications in angles and heatmap in a homozygous fz_{p21} null pupal wing. Scale bars represent 10µm. $p<0.0001$ (Chi-square test) (vs *WT* control; Supplementary Figure 2**s**). Scale bars represent 10µm.

(**s-w**) Co-staining of centrioles (Sas4 in green; monochrome channel in **u**), the junctional marker Fmi (in red; monochrome channel in **t**),) and the actin based hairs labeled with phalloidin (in blue; monochrome channel in **w**) in a *dpp driven* over-expression of Fz. Scale bars represent 10µm.

(**y-z**) Centriole distribution quantifications in angles and heatmap in Fz overexpression experiments. Statistical analysis: $p<0.0001$(Chi-square test) (vs. *WT* control; Supplementary Figure 2**s**). Scale bars represent 10µm.

Supplementary Figure 4: Actin and acetylated tubulin in pupal wings

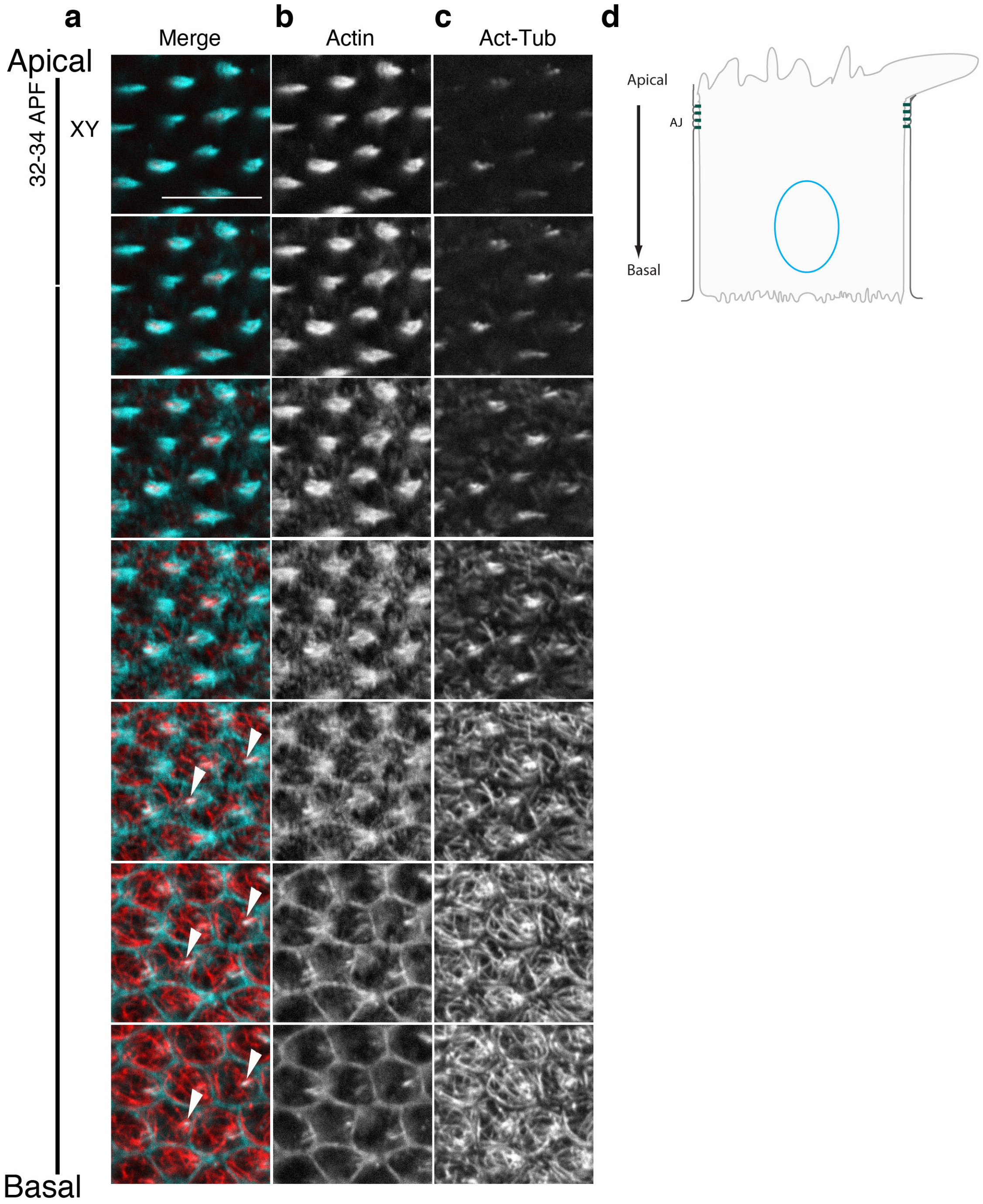

(**a-c**) Several XY sections from the same wing area depicted in Figure 3, showing actin and acetylated tubulin in more basal planes (actin labeled with Rhodamine phalloidin in red and acetylated tubulin in cyan). Arrowheads point towards the base of the hair, note overlap of Actin and Ac-tubulin at the base of hairs. Scale bars represent 10μm.

(**d**) Illustration of an epithelial cell in a Z-view.

Supplementary Figure 5: Centrioles position relative to actin

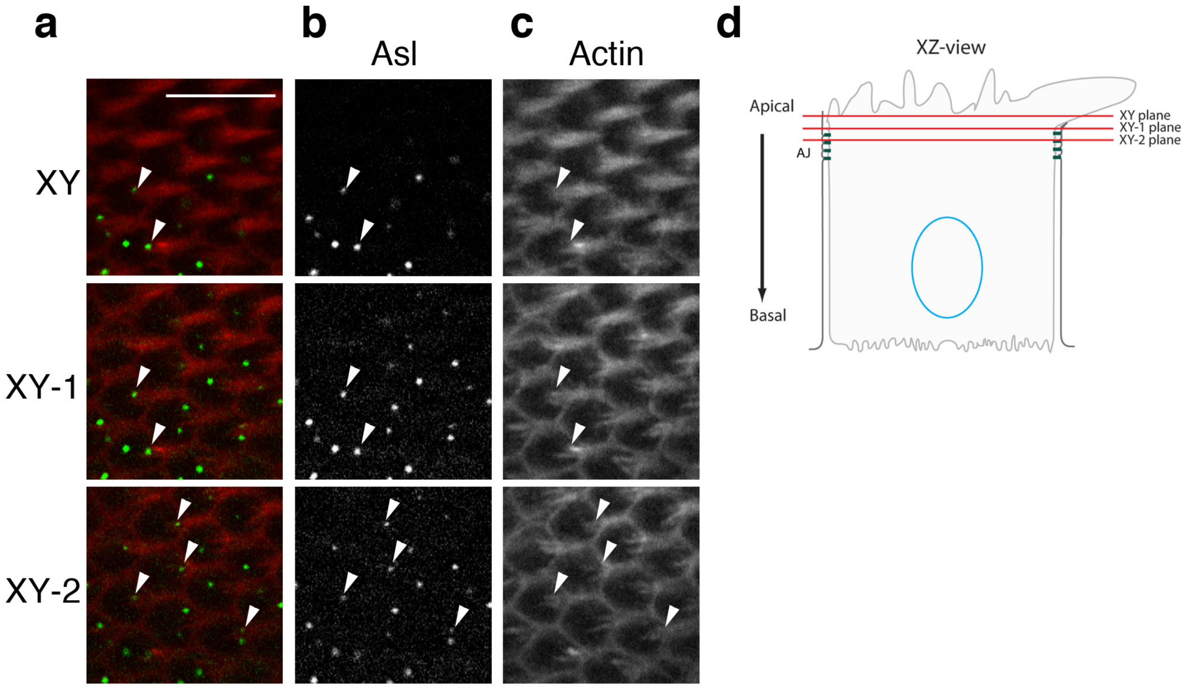

(**a-c**) Three XY section from the same wing area showing centrioles (**b**, in green) position relative to actin (**c**, in red) in WT pupae wings. Arrowheads point to centrioles that are associated with the base of the hairs. Scale bars represent 10μm.

(**d**) Illustration of an epithelial cell in a Z-view.

Supplementary Figure 6: Actin and acetylated tubulin in PCP LOF and GOF

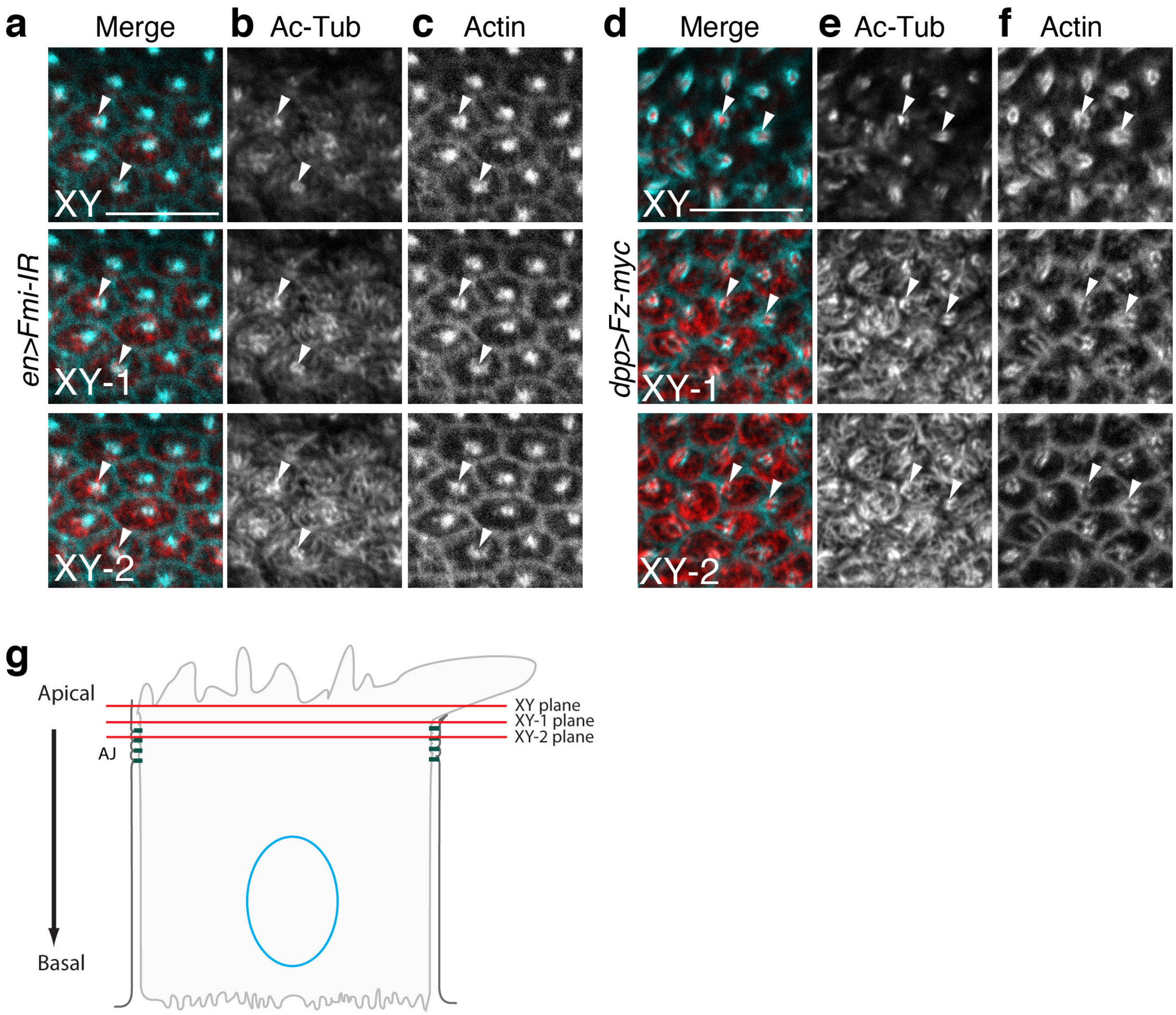

(**a-c**) Three XY sections of the same area showing actin and acetylated tubulin in *en>Fmi IR* wing cells (actin labeled with Rhodamine phalloidin depicted in red and acetylated tubulin in cyan). Note juxtaposition of Ac-Tub and Actin staining (arrowheads) at base of hairs (central position in this PCP LOF background)

(**d-f**) Three XY sections of the same area showing actin and acetylated tubulin in *dpp>Fz* over-expression (PCP gain-of-function; actin labeled with Rhodamine phalloidin in red and acetylated tubulin in cyan). Note juxtaposition of Ac-Tub and Actin; arrowheads point to acetylated-tubulin that is associated with the base of the actin hair (immediately surrounded by Actin). Scale bars represent 10µm.

(**g**) Illustration of an epithelial cell in a Z-view to indicate planes of optical sections in **a-d**.

Supplementary Figures 7: Fmi polarity vectors in Gain or loss of centrioles number

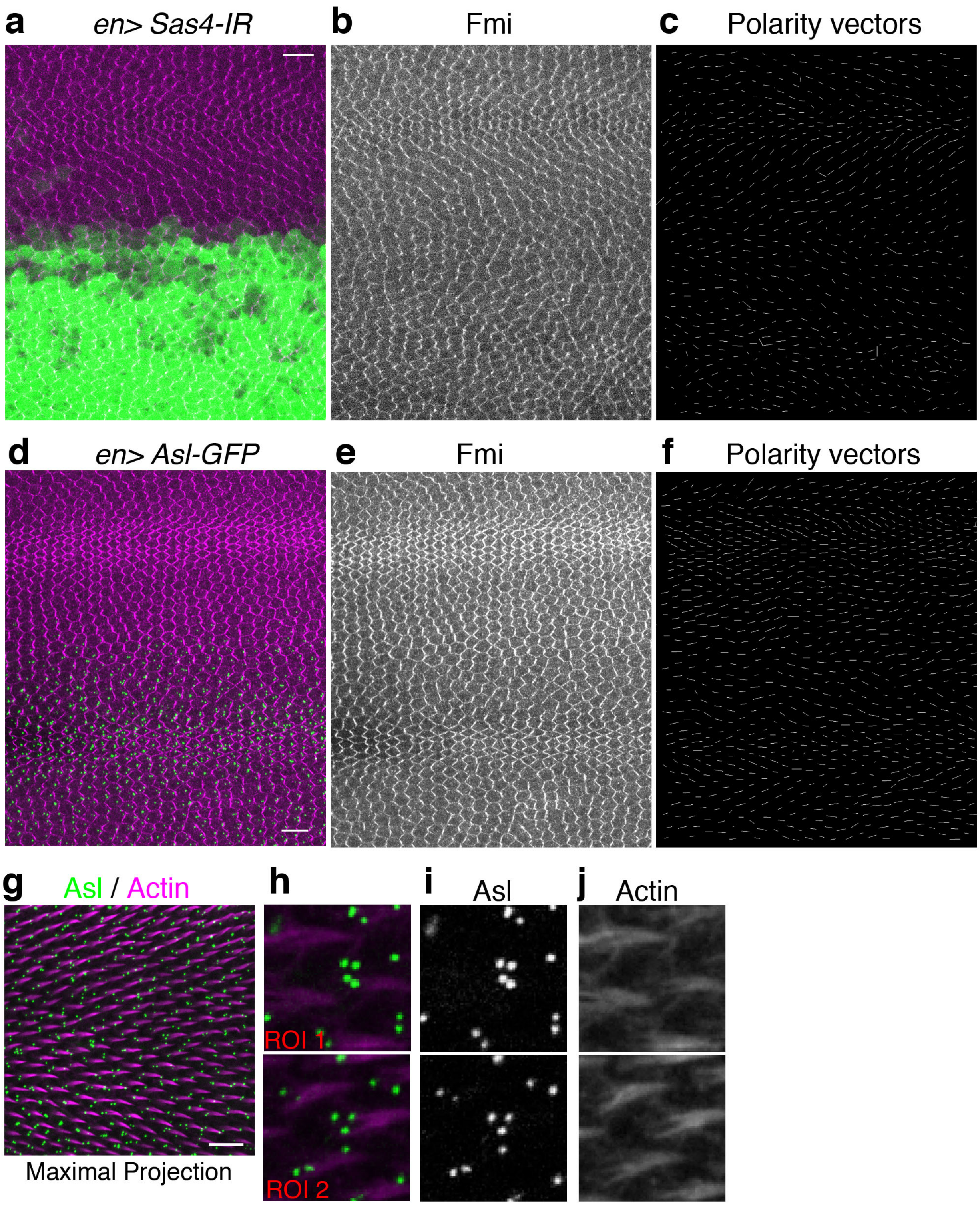

(**a-c**) An example of a pupal wing with *en>* driven knock-down of Sas4 showing the engrailed domain (**a**) in green, Fmi staining in magenta (**a** and **b** monochrome channel) and the calculated polarity vectors (nematic order) based on Fmi staining (**c**). Note largely normal polarity vectors as compared to *wild type*. Scale bars represent 10μm.

(**d-f**) An example of a pupal wing with en> driven over-expression of Asl showing Asl (**d**) in green, Fmi staining in magenta (**d** and **e,** monochrome channel) and the calculated polarity vectors (nematic order) based on Fmi staining (**f**). Again, note largely normal polarity vectors as compared to *wild type*. Scale bars represent 10μm.

(**g**) Maximal projection of a pupal wing over-expressing Asl-GFP, showing the relative position of Asl-positive centrioles to the actin based hairs.

(**h-j**) Two regions of interest (ROI 1 and ROI 2) from the field shown in (**g**) at high magnification, highlighting the positioning of multiple centrioles (**i**; green in **h**) relative to polymerized actin (**j**, magenta in **h**) in Asl over-expressing pupal wings. Scale bars represent 10μm.

Supplementary Figure 8: Fmi polarity vectors in *sas4* null allele)

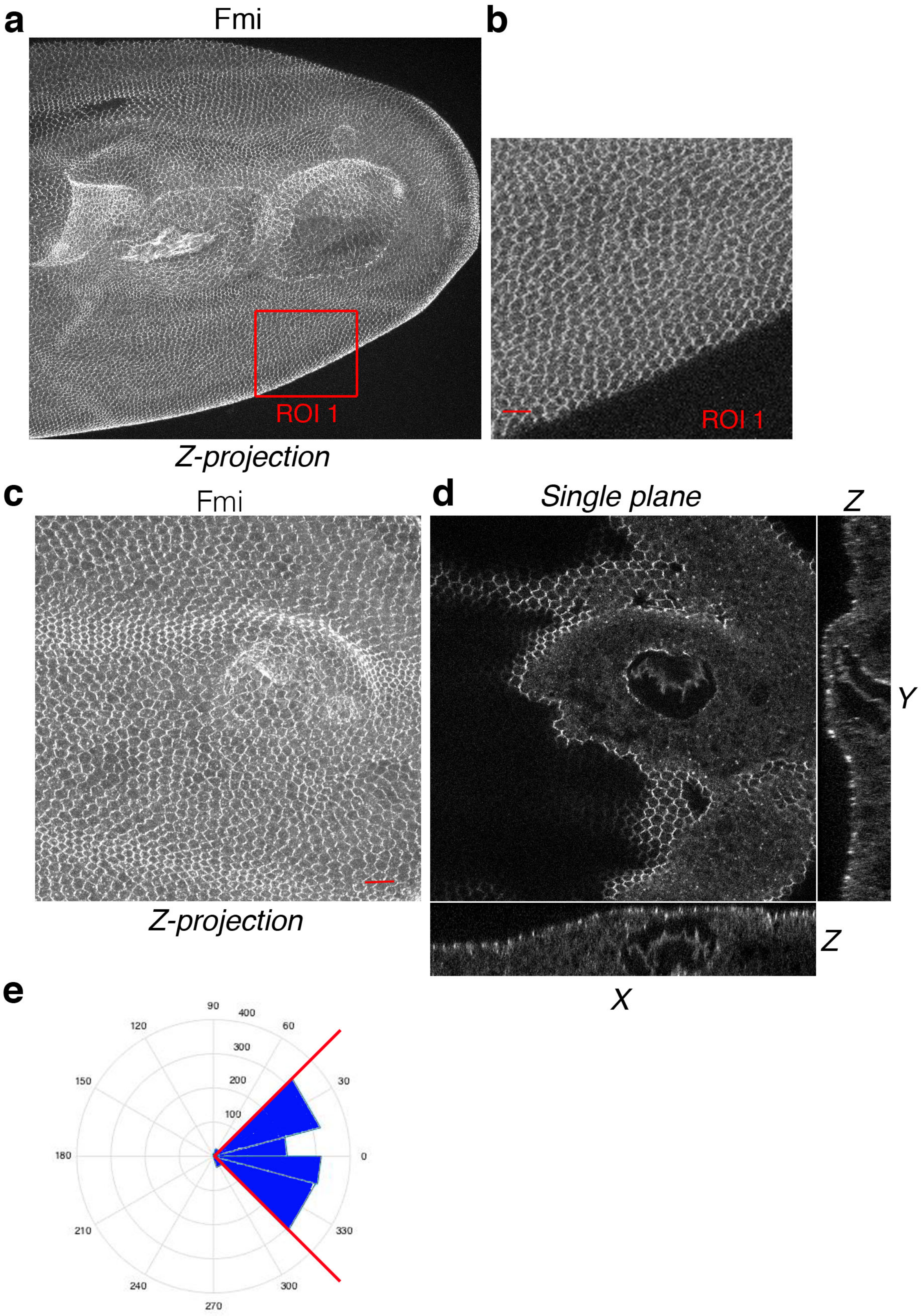

(**a-b**) An example of a whole *sas4 S2214* null pupal wing (shown in monochromo channel)

and stained for Fmi in **a**; **b** displays high magnification of region of interest (ROI1 boxed in **a**). Note the bulging tissue seen in the center of the wing is due to the compensatory proliferation (overgrowth) and not due to PCP defects. Scale bars represent 10µm.

(**c-d**) A second example of a *sas4* $_{S2214}$ pupal wing showing a projection (in monochromo channel) of Fmi staining in **c** and a single plane in XY, XZ and YZ sections of the same wing area depicted in **d**. Note in both figure panels, **b** and **c,** that Fmi staining is largely normal, and in **c** a relatively small tissue overgrowth. Scale bars represent 10µm.

(**e**) Quantification of angle distribution of polarity vectors from the *sas4* $_{S2214}$ null pupal wing in **c**; note that PCP angle distribution is basically like in wild-type and contained within the distally oriented quadrant (compare to other figures)
